# Temporal relationship between body mass index and uric acid and their joint impact on blood pressure in children and adults: the Bogalusa Heart Study

**DOI:** 10.1038/s41366-021-00810-9

**Published:** 2021-04-06

**Authors:** Miaoying Yun, Tao Zhang, Shengxu Li, Xuan Wang, Lijun Fan, Yinkun Yan, Lydia Bazzano, Jiang He, Wei Chen

**Affiliations:** 1 Center on Translational Neuroscience, College of Life and Environment Sciences, Minzu University of China, Beijing, China; 2 Department of Biostatistics, School of Public Health, Cheeloo College of Medicine, Shandong University, Jinan, Shandong China; 3 Children’s Minnesota Research Institute, Children’s Hospitals and Clinics of Minnesota, Minneapolis, MN USA; 4 Department of Epidemiology, School of Public Health, Harbin Medical University, Harbin, Heilongjiang China; 5 Institute for Iodine Deficiency Disorders Prevention and Control, Center for Endemic Disease Control, Chinese Center for Disease Control and Prevention, Harbin Medical University, Harbin, Heilongjiang China; 6 Department of Epidemiology, School of Public Health and Tropical Medicine, Tulane University, New Orleans, LA USA; 7 Beijing Children’s Hospital, Capital Medical University, National Center for Children’s Health, Beijing, China

**Keywords:** Epidemiology, Risk factors

## Abstract

**Objective:**

This study aimed to examine the temporal relationship between body mass index (BMI) and uric acid (UA), and their joint effect on blood pressure (BP) in children and adults.

**Methods:**

The longitudinal cohorts for temporal relationship analyses consisted of 564 and 911 subjects examined twice 5–14 years apart from childhood to adulthood. The cross-sectional cohorts for mediation analyses consisted of 3102 children and 3402 nondiabetic adults. Cross-lagged panel analysis models were used to examine the temporal relationship between BMI and UA, and mediation analysis models the mediation effect of UA on the BMI–BP association.

**Results:**

After adjusting for age, race, sex and follow-up years in children, and additionally smoking and alcohol drinking in adults, the path coefficients (standardized regression coefficients) from baseline BMI to follow-up UA (0.145 in children and 0.068 in adults) were significant, but the path coefficients from baseline UA to follow-up BMI (0.011 in children and 0.016 in adults) were not. In mediation analyses, indirect effects through UA on the BMI-systolic BP association were estimated at 0.028 (mediation effect = 8.8%) in children and 0.033 (mediation effect = 13.5%) in adults (*P* < 0.001 for both). Direct effects of BMI on systolic BP (0.289 in children and 0.212 in adults) were significant. The mediation effect parameters did not differ significantly between Blacks and Whites.

**Conclusions:**

Changes in BMI precede alterations in UA, and the BMI–BP association is in part mediated through BMI-related increase in UA both in children and in adults. These findings have implications for addressing mechanisms of obesity hypertension beginning in early life.

## Introduction

Uric acid (UA) is a major oxidation product of purine metabolism. Once it enters vascular smooth muscle cells and adipocytes, UA has detrimental effects, such as dampened endothelial function, induced platelet aggregation and chronic systemic inflammation [1–4]. Evidence from longitudinal epidemiologic studies shows that hyperuricemia is linked to future development of cardiovascular disease, hypertension, and diabetes [5–8]. The Bogalusa Heart Study has shown that increased UA levels in childhood are associated with higher risk of adult hypertension [7]. Obesity, a major risk factor for hypertension, is closely correlated with elevated UA levels in both children and adults [9–11].

Despite the strong association between obesity and hyperuricemia, their causal sequence remains largely unsettled. Studies have indicated that obesity leads to hyperuricemia [12, 13], but others have demonstrated a causal role of increased UA levels in developing obesity [8, 14–18]. Further, although there is a large body of literature regarding the relationship between obesity, hyperuricemia and hypertension, convincing evidence is lacking from the general population on the causal sequence between changes in body weight and UA and the implication of this causal sequence on the development of hypertension. In this study we aimed to examine the temporal relationship between body weight and UA, and their joint effect on blood pressure (BP) levels in children and adults. These aims were achieved by leveraging existing longitudinal and cross-sectional cohorts of children and adults in the Bogalusa Heart Study.

## Methods

### Study cohort

The Bogalusa Heart Study, established in a semi-rural, Black–White (65% White and 35% Black) community in Bogalusa, Louisiana since 1973, focuses on the natural history of cardiovascular disease and its risk factors beginning in childhood [19]. Between 1986 and 2016, one child survey in 1986–87 (age = 5–19 years, *n* = 3102) and three adult surveys in 1995–2000 (age = 20–30 years, *n* = 1457), 2001–04 (age = 31–42 years, *n* = 1506) and 2009–16 (age = 43–56 years, *n* = 1739) were conducted in Bogalusa. These nondiabetic participants had data on serum UA, body mass index (BMI), BP, cigarette smoking, alcohol drinking and demographics. By linking children and adults in the 1995–2000 survey, 564 participants were identified who were examined twice 7–14 years apart. By linking the 2001–04 and 2009–16 adult surveys, 911 nondiabetic adult participants were identified who were examined twice 5–14 years apart. By combining the three adult surveys, 3402 nondiabetic participants who were examined once were identified as a cross-sectional cohort of adults. The two longitudinal cohorts from childhood to adulthood were used for temporal relationship analysis between BMI and UA. The cross-sectional cohorts of children (*n* = 3102) and adults (*n* = 3402) were used for mediation analysis. The cohorts and analysis design are described in the flow chart in Supplementary Fig. S1.

All participants or their legal guardians gave informed consent. Study protocols were approved by the institutional review board of the Tulane University Health Sciences Center.

### Measurements

Both height and weight were measured twice, and their mean values were used for analysis. BMI was calculated as weight in kilograms divided by height in meters squared. Serum UA levels were measured as part of SMA20 by the multichannel Olympus Au-5000 Analyzer (Olympus) with the uricase method. Systolic and diastolic blood pressure (SBP and DBP) were recorded at 8:00–10:00 a.m. on the right arm of subjects in a relaxed, sitting position by two trained nurses (three replicates each). The mean values of the 6 readings were used for analysis. Hypertension in adults was defined as SBP/DBP ≥ 130/80 mm Hg or taking antihypertensive medications [20]. When BP was analyzed as a continuous variable, the mean BP values of hypertensive patients taking medications were adjusted by adding 10 mm Hg to SBP and 5 mm Hg to DBP based on average treatment effects [21].

### Statistical methods

**Fig. 1:**
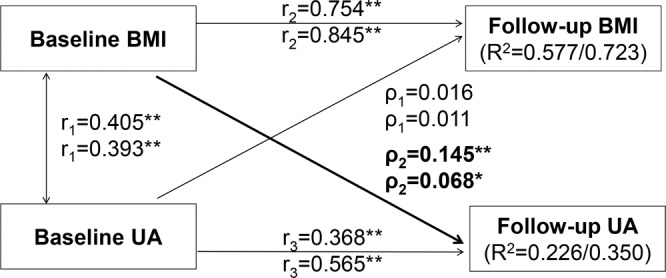
Cross-lagged path analysis models of BMI and uric acid in follow-up periods from childhood to adulthood (upper) and during adulthood (lower). BMI = body mass index; UA = uric acid. *ρ*_1_, *ρ*_2_ = cross-lagged path coefficients; *r*_1_ = synchronous correlations; *r*_2_, *r*_3_ = tracking correlations; *R*^2^ = variance explained; Comparative fit index (CFI) = 0.92/0.96; Root mean square residual (RMR) = 0.02/0.01. Coefficients different from 0: *P < 0.05, **P < 0.01.

To examine the temporal relationship between BMI and UA levels, we used the cross-lagged panel analysis, a form of path analysis that simultaneously examines reciprocal and longitudinal relationship among intercorrelated variables [22]. In the current study, BMI and UA levels measured at the same two time points (both baseline and follow-up surveys) made the cross-lagged panel an excellent method to disentangle their temporal relationship. In an overall, simplified conceptual model (Fig. 1), the path with *ρ*_1_ describes the effect of baseline UA on follow-up BMI, and the path with *ρ*_2_ describes the effect of baseline BMI on follow-up UA levels. Before the cross-lagged path analysis, both baseline and follow-up values of BMI and UA were controlled for age in children and additionally smoking and alcohol drinking in adults by regression residual analyses and then standardized by Z-transformation (mean = 0, SD = 1) in separate race-sex groups. The path coefficients (*ρ*_1_ and *ρ*_2_) in Fig. 1 were estimated simultaneously, with additional adjustment for follow-up years, in a structural equation model using R package *lavaan*. Root mean square residual (RMR) and comparative fit index (CFI) were used to scrutinize the validity of model fitting, with RMR > 0.90 and CFI < 0.05 suggesting good model fitting. A significant path coefficient (*ρ*_1_ or *ρ*_2_) suggests their respective directionality of the pathways. We also performed stratified analyses by hypertension status, race, sex, and follow-up duration groups. The differences in ρ_1_ and ρ_2_ between the respective strata were tested by using Fisher’s *Z*-test as described previously [23].

**Fig. 2:**
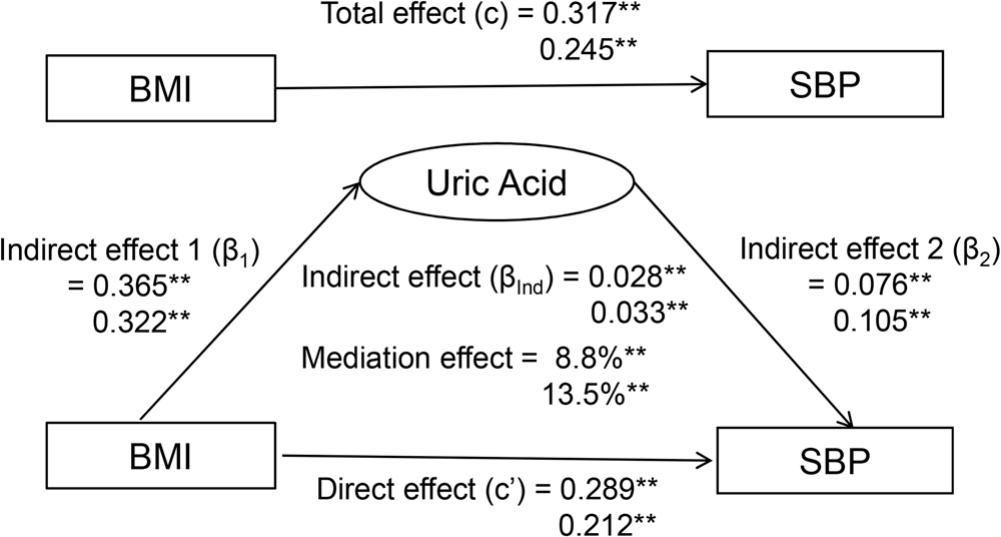
Mediation effect model of uric acid on the BMI-SBP association in children (upper) and adults (lower). BMI = body mass index; SBP = systolic blood pressure. *β*, *c* and *c*′ are standardized regression coefficients; *c* = total effect; *c*′ = direct effect; *β*_1_ = indirect effect 1; *β*_2_ = indirect effect 2; *β*_Ind_ = total indirect effect (*β*_1_ × *β*_2_). Coefficients different from 0: **P < 0.01. $$\begin{array}{l}{\mathrm{Indirect}}\,{\mathrm{effect}}\left( {{\upbeta }}_{{\mathrm{Ind}}} \right) = {\upbeta}_1 \times {\upbeta}_2 = 0.365 \times 0.076 = 0.028\\ 0.322 \times 0.105 = 0.033\end{array}$$ $$\begin{array}{l}{\mathrm{Total}}\,{\mathrm{effect}}\left( {\mathrm{c}} \right) = {\mathrm{Direct}}\,{\mathrm{effect}}\left( {c\prime } \right) + {\mathrm{Indirect}}\,{\mathrm{effect}}\left( {{\upbeta }}_{{\mathrm{Ind}}} \right) = 0.289 + 0.028 = 0.317\\ 0.212 + 0.033 = 0.245\end{array}$$ $$\begin{array}{l}{\mathrm{Mediation}}\,{\mathrm{effect}} = \left( {{\upbeta}_1 \times {\upbeta}_2} \right)/{\mathrm{c}} \times 100 = 0.028/0.317 \times 100 = 8.8\% \\ 0.033/0.245 \times 100 = 13.5\% \end{array}$$.

The causal mediation analysis model [24] in Fig. 2 was constructed to examine the mediation effect of UA on the association between BMI and SBP. BMI was the predictor variable (*X*), UA the mediator (*M*), and SBP the outcome variable (*Y*). The mediation effect analyses were conducted using multivariable regression models, with age, race and sex in children, and additionally smoking and alcohol drinking in adults included for adjustment. In this study, we performed the mediation analyses in the following four steps: (1) regressing SBP on BMI (Model *Y* = *cX*) where c is total effect; (2) regressing UA levels on BMI (Model *M* = *β*_1_ *X*) where *β*_1_ is indirect effect 1; (3) regressing SBP on UA levels controlling for BMI (Model *Y* = *β*_2_*M* + c’*X*) where *β*_2_ is indirect effect 2, and *c*′ is direct effect; and (4) calculating mediation effect as (*β*_1_ × *β*_2_)/*c*. Differences in mediation effect parameters between subgroups were tested using interaction regression models by including interaction terms of BMI- or UA-group variables. Mediation analyses with BP as a continuous variable and with hypertension as a dichotomous variable were conducted using R package *lavaan*.

## Results

Table 1 summarizes descriptive data of two longitudinal cohorts for temporal relationship analyses by race and sex. For children in longitudinal cohort I, there were significant race and sex differences in UA levels. For adults in longitudinal cohort I, black females had highest BMI among the four groups; there were significant sex differences in levels of UA and DBP; SBP had significant race and sex differences. In longitudinal cohort II, black females had highest BMI among the four groups at both baseline and follow-up; SBP and DBP had significant race and sex differences; UA at baseline and follow-up had significant sex differences for both races.

**Table 1:** Characteristics of the longitudinal cohorts by race and sex.

Characteristic	White		Black		P for race difference	
	Male	Female	Male	Female	Male	Female
Longitudinal cohort I	*N* = 140	*N* = 183	*N* = 98	*N* = 143		
Children at baseline						
Age (year)	13.6 (3.3)	13.4 (3.1)	12.7 (3.8)	13.0 (3.6)	0.046	0.317
BMI (kg/m^2^)	21.3 (4.6)	21.4 (5.4)	20.6 (5.7)	21.6 (5.2)	0.988	0.339
Uric acid (mg/dL)	5.1 (1.4)	4.1 (1.1)**	4.4 (1.5)	3.7 (1.2)**	0.001	0.011
SBP (mmHg)	105 (11)	104 (10)	104 (14)	103 (11)	0.126	0.984
DBP (mmHg)	62 (9)	65 (8)**	62 (10)	63 (10)	0.170	0.121
Adults at follow-up						
Age (year)	25.4 (4.2)	24.4 (3.6)*	24.1 (4.3)	24.5 (4.0)	0.018	0.801
Smokers, *n* (%)	41 (29.3)	56 (30.6)	27 (27.6)	20 (14.0)**	0.771	<0.001
Drinkers, *n* (%)	51 (36.4)	29 (15.9)**	45 (45.9)	19 (13.3)**	0.142	0.517
BMI (kg/m^2^)	28.3 (6.2)	27.9 (8.0)	27.9 (6.8)	31.2 (8.7)**	0.955	<0.001
Uric acid (mg/dL)	6.2 (1.2)	4.3 (1.1)**	5.8 (1.3)	4.3 (2.4)**	0.166	0.811
SBP (mmHg)	116 (9)	108 (9)**	118 (12)	110 (12)**	0.027	0.043
DBP (mmHg)	76 (7)	71 (7)**	76 (9)	72 (11)**	0.613	0.708
Longitudinal cohort II	*N* = 276	*N* = 379	*N* = 89	*N* = 167		
Adults at baseline						
Age (year)	34.8 (4.7)	34.4 (4.9)	34.4 (4.3)	34.0 (5.1)	0.342	0.403
Smokers, *n* (%)	74 (26.8)	100 (26.4)	43 (48.3)	48 (28.7)**	<0.001	0.568
Drinkers, *n* (%)	94 (34.1)	58 (15.3)**	51 (57.3)	35 (21.0)**	<0.001	0.105
BMI (kg/m^2^)	28.0 (4.9)	27.0 (6.6)*	28.1 (6.9)	31.1 (8.1)**	0.995	<0.001
Uric acid (mg/dL)	6.0 (1.2)	4.2 (1.1)**	6.1 (1.3)	4.2 (1.3)**	0.405	0.645
SBP (mmHg)	116 (10)	109 (10)**	122 (14)	117 (15)**	<0.001	<0.001
DBP (mmHg)	78 (8)	74 (8)**	81 (11)	78 (10)*	<0.001	<0.001
Adults at follow-up						
Age (year)	45.6 (6.2)	45.1 (6.5)	44.5 (5.8)	45.3 (6.6)	0.132	0.765
Smokers, *n* (%)	66 (23.9)	104 (27.4)	43 (48.3)	45 (27.0)**	<0.001	0.905
Drinkers, *n* (%)	125 (45.3)	113 (29.8)**	44 (49.4)	27 (16.2)**	0.495	<0.001
BMI (kg/m^2^)	30.0 (5.5)	29.4 (7.2)	29.6 (8.3)	34.4 (8.8)**	0.579	<0.001
Uric acid (mg/dL)	6.2 (1.3)	4.6 (1.3)**	6.4 (1.6)	4.9 (1.5)**	0.217	0.013
SBP (mmHg)	122 (12)	115 (13)**	130 (16)	124 (20)**	<0.001	<0.001
DBP (mmHg)	80 (9)	75 (9)**	85 (11)	81 (12)**	<0.001	<0.001

Data are presented in means (SD) or *n* (%).

Sex difference within racial groups: *p < 0.05; **p < 0.01.

*BMI* body mass index, *SBP* systolic blood pressure, *DBP* diastolic blood pressure.

Table 2 shows characteristics of study variables of the cross-sectional cohorts of children and adults for mediation analyses by race and sex. There were significant race and sex differences in UA levels in children and adults except for race difference in male adults. SBP and DBP had significant race and sex differences in adult cohort. There were significant race and sex differences in prevalence of hypertension except for race difference in males.

**Table 2:** Characteristics of the cross-sectional cohorts of children and adults by race and sex.

Characteristic	White		Black		*P* for race difference	
	Male	Female	Male	Female	Male	Female
Children cohort						
*N*	953	885	648	616		
Age (year)	11.0 (3.9)	11. 0 (3.5)	11.5 (3.8)	11.4 (3.7)	0.018	0.015
BMI (kg/m^2^)	19.2 (4.2)	19.6 (4.9)*	19.4 (4.7)	19.7 (4.7)	0.850	0.344
Uric acid (mg/dL)	4.5 (1.4)	4.1 (1.1)**	4. 1 (1.4)	3.6 (1.0)**	<0.001	<0.001
SBP (mmHg)	100 (11)	100 (10)	102 (12)	101 (11)*	0.026	0.934
DBP (mmHg)	58 (9)	60 (10)**	59 (10)	60 (11)	0.596	0.188
Adult cohort						
*N*	1012	1209	509	672		
Age (year)	36.9 (11.0)	37.1 (10.9)	34.3 (11.5)	35.1 (11.8)*	<0.001	<0.001
Smokers, *n* (%)	349 (34.5)	401 (33.2)	197 (38.7)	161 (24.0)**	0.105	<0.001
Drinkers, *n* (%)	366 (36.2)	251 (20.8)**	201 (39.5)	96 (14.3)**	0.206	<0.001
BMI (kg/m^2^)	28.6 (6.0)	28. 0 (7.4)**	28.2 (7.3)	31.4 (8.7)**	0.862	<0.001
Uric acid (mg/dL)	6.1 (1.3)	4.5 (1.2)**	6.2 (1.5)	4.6 (1.7)**	0.307	0.010
SBP (mmHg)	119 (13)	112 (12)**	124 (17)	118 (18)**	<0.001	<0.001
DBP (mmHg)	79 (10)	74 (9)**	80 (13)	77 (12)**	<0.001	<0.001
Hypertension, *n* (%)	478 (47.2)	367 (31.1)**	258 (50.7)	296 (44.1)*	0.203	<0.001

Data are presented in means (SD) or *n* (%).

Sex difference within racial groups: *p < 0.05, **p < 0.01.

*BMI* body mass index, *SBP* systolic blood pressure, *DBP* diastolic blood pressure.

For cross-lagged panel analyses of BMI and UA (Fig. 1), with adjustment for age, race, sex and follow-up years in children, and additionally smoking and alcohol drinking in adults, the path coefficients from baseline BMI to follow-up UA were significant in both childhood and adulthood (*ρ*_2_ = 0.145 in children and 0.068 in adults), but the path coefficients from baseline UA to follow-up BMI (*ρ*_1_ = 0.016 in children and 0.011 in adults) were not. The tracking correlations of BMI from baseline to follow-up in children and adults were higher than the tracking correlations of UA and the synchronous correlation between BMI and UA at baseline. According to CFI and RMR, the models fit well with the observed data (CFI = 0.92 for children and 0.96 for adults; RMR = 0.02 for children and 0.01 for adults).

Results for subgroup analyses in two longitudinal cohorts are shown in Supplementary Tables S1 and S2. In Supplementary Table S1, the path coefficients from baseline UA to follow-up BMI (*ρ*_1_) in subgroups were all nonsignificant during the period from childhood to adulthood; the path coefficients from baseline BMI to follow-up UA (*ρ*_2_) in subgroups were all significant except for normotensives and Blacks. In Supplementary Table S2, only the path coefficient from baseline BMI to follow-up UA (*ρ*_2_) in Whites was significant. All the path coefficients (ρ_1_ and ρ_2_) did not differ significantly between subgroups. The model fitting parameters ranged from 0.01 to 0.08 for RMR and from 0.86 to 1.00 for CFI, indicating a relatively good fit to the observed data.

With the above results suggesting that BMI at baseline leads to changes in UA levels at follow-up, we performed mediation analyses in both children and adults (Fig. 2). With adjustment for age, race and sex in children, and additionally smoking and alcohol drinking in adults, total effects of BMI on SBP in children/adults (*c* = 0.317/0.245) were significant without UA in the model. The overall indirect effects of BMI on SBP through UA were estimated at *β*_Ind_ = 0.028 in children and 0.033 in adults (*p* < 0.01 for both). The mediation effects of UA on the association between BMI and SBP were 8.8% in children and 13.5% in adults. The direct effects of BMI on SBP (*c*′ = 0.289 in children and 0.212 in adults) were significant. In subgroup mediation analyses (Supplementary Table S3), mediation parameters did not differ significantly between race groups for children and adults; sex differences were significant in total effect, indirect effect 1 and direct effect in children, and total effect and direct effect in adults. The mediation effect of UA in relation to DBP was not significant in children (Supplementary Fig. S2). Similar mediation results were observed if hypertension was used as a dichotomous outcome (Supplementary Fig. S3).

## Discussion

In the current study, we first established the temporal relationship between BMI and UA in two longitudinal cohorts and then examined the mediation effect of UA on the BMI–BP association in two cross-sectional samples from the Bogalusa Heart Study. The central findings are that changes in baseline BMI precede changes in follow-up UA levels and that the association between BMI and BP is partly mediated by BMI-related alterations in UA levels, which were noted consistently in children and adults.

Obesity is often accompanied by hyperuricemia, and they are both independent risk factors of essential hypertension [5–7, 25–27]. Although increased UA is generally thought to be the result of obesity/overweight rather than its cause, there is evidence that the relationship between BMI and UA may be bi-directional based on pathophysiological and metabolic mechanisms [8, 12–18]. Human and animal studies have shown that hypoxanthine, the precursor of UA in purine catabolism, is secreted from human adipose tissue, and high production of hypoxanthine in adipocytes leads to overproduction of UA [12, 13]. On the other hand, fructose-mediated generation of UA has a causal role in weight gain [8]. Longitudinal epidemiologic studies have reported that increased UA levels at baseline significantly predict development of incident obesity and other metabolic syndrome components [14–17]. A recent longitudinal study has demonstrated that the relationship between UA and BMI is bi-directional although the UA-to-BMI path is stronger than the BMI-to-UA path [18]. In the current study, we adopted a powerful cross-lagged panel analysis method and found that changes in BMI preceded alterations in UA at follow-up. Such a unidirectional causal pathway was consistent in the two longitudinal study cohorts followed from childhood to young adulthood and from young adulthood to midlife. These findings provided fresh and strong evidence that obesity is more likely to be the cause, not a consequence, of elevated UA levels.

One of the most common comorbidities associated with obesity is essential hypertension. Epidemiology studies indicate that 65–75% of essential hypertension is due to overweight or obesity [25]. There are multiple potential mechanisms linking obesity to hypertension, including dietary factors, metabolic, endothelial, vascular and renal dysfunction, neuroendocrine imbalances, sodium retention, and maladaptive immune and inflammatory responses [26, 27]. Despite considerable progress towards unraveling the complex interactions between these factors, it is unknown whether increased UA levels as a mediating factor link excess adiposity with elevated BP.

The temporal sequence of BMI-to-UA as shown in Fig. 1 indicates that UA as a third variable is not a confounder for the BMI–BP association by definition [28] because it is intermediate in the causal pathway between the predictor (BMI) and the outcome (BP). In this study, we built the mediation model for the BMI–BP association with UA as the mediator. The mediation effect of UA was estimated as 8.8% in children and 13.5% in adults, suggesting that high BMI-induced increase in UA is one of the mechanisms underlying the development of obesity hypertension. Furthermore, the significant direct effects of BMI on BP in children and adults underscore the importance of other known and/or unknown mechanisms linking obesity and hypertension.

Blacks outpace other racial/ethnic groups in the United States in terms of prevalence, early onset and severity of hypertension [29]. Blacks had higher BP levels and a faster rate of change in BP than Whites even in childhood [30]. Despite the marked Black-White difference in BP in adults, BMI and UA in female adults observed in this study, the path and mediation parameters did not differ significantly between black and white adults. Our study showed significant sex differences in UA (males > females) which was consistent in children and adults, but the path coefficients for BMI and UA were not significantly different between sex groups. In mediation analyses, total and direct effects showed significant sex differences with different directions in children and adults. To date, no data are available in this regard for reference and comparison. Further research work remains to be performed to confirm the findings of the current study, particularly for subgroup analyses.

Most studies that evaluate correlation between BMI and UA have been cross-sectional [10, 11] and thus limit inferences regarding cause and effect. The present community-based longitudinal study cohorts provided an opportunity to examine the temporal relationship between BMI and UA during two growth periods. This study also has limitations. First, individuals under antihypertensive pharmacological medications would represent a subgroup having the highest BP levels without treatment. We included this subgroup of adults by adjusting their measured BP values based on the average effect of medications. This adjustment method may result in bias in analyses to some extent in the adult cohort. Second, the sample size of the black group is relatively small for race- and sex-specific temporal relationship analyses. Subgroup analyses in large-scale longitudinal cohorts are needed to examine race and sex differences in temporal sequence parameters.

In summary, we first demonstrated that changes in BMI led to alterations in UA levels in two longitudinal cohorts using the cross-lagged analysis models. With the unidirectional path from baseline BMI to follow-up UA established, we found in mediation analyses that increase in UA levels had a significant mediation effect on the BMI–BP association in both children and adults, indicating that obesity-related hyperuricemia is one of the mechanisms underlying the development of obesity hypertension. Findings from the current study will help better our understanding of the mechanisms for hypertension and improve the early life intervention strategies by targeting the cause factors and related mediators to reduce the hypertension risk in adults.

## Supplementary information

Supplemental Materials

## Data Availability

The data that support the findings of this study are available from the corresponding author upon reasonable request. Part of the Bogalusa Heart Study data are publicly available at https://biolincc.nhlbi.nih.gov/studies/bhs.
