# Supplementary material for: Temporal relationship between body mass index and uric acid and their joint impact on blood pressure in children and adults: the Bogalusa Heart Study
Source: Int J Obes (Lond). 2021 Apr 6;45(7):1457–63. doi: 10.1038/s41366-021-00810-9 (PMC8236402; doi:10.1038/s41366-021-00810-9)

| **Table S1.** Cross-lagged path coefficients between BMI and UA from childhood to young adulthood by subgroups | | | | | | | |
| --- | --- | --- | --- | --- | --- | --- | --- |
|  | Path Coefficients | | | |  | Goodness-of-fit | |
|  | UA → BMI  (ρ_1_) |  | BMI → UA  (ρ_2_) | P^a^ |  | RMR | CFI |
| Adult HTN group |  |  |  |  |  |  |  |
| NTN (n=437) | 0.012 |  | 0.103* | 0.178 |  | 0.03 | 0.98 |
| HTN (n=127) | 0.009 |  | 0.157 | 0.190 |  | 0.06 | 0.86 |
| P^b^ | 0.976 |  | 0.589 |  |  |  |  |
| Race |  |  |  |  |  |  |  |
| White (n=323) | 0.056 |  | 0.219** | 0.035 |  | 0.01 | 0.96 |
| Black (n=241) | -0.028 |  | 0.051 | 0.388 |  | 0.02 | 0.92 |
| P^b^ | 0.326 |  | 0.045 |  |  |  |  |
| Sex |  |  |  |  |  |  |  |
| Male (n=238) | -0.007 |  | 0.128* | 0.141 |  | 0.08 | 0.87 |
| Female (n=326) | 0.039 |  | 0.156** | 0.133 |  | 0.01 | 0.90 |
| P^b^ | 0.591 |  | 0.739 |  |  |  |  |
| Follow-up years |  |  |  |  |  |  |  |
| 7~10 years (n=231) | -0.005 |  | 0.202** | 0.025 |  | 0.04 | 0.98 |
| 11~14 years (n=333) | 0.040 |  | 0.103* | 0.416 |  | 0.01 | 1.00 |
| P^b^ | 0.601 |  | 0.239 |  |  |  |  |
| ρ =standardized regression coefficient; RMR=root mean square residual; CFI=comparative fit index; UA=uric acid; BMI=body mass index; NTN=normotension; HTN= hypertension (defined as SBP/DBP>=130/80 mmHg or taking medications in adults)  a, p value for difference between ρ_1_ and ρ_2_  b, p value for difference between subgroups | | | | | | | |

| **Table S2.** Cross-lagged path coefficients between BMI and UA during adulthood by subgroups | | | | | | | |
| --- | --- | --- | --- | --- | --- | --- | --- |
|  | Path Coefficients | | | |  | Goodness-of-fit | |
|  | UA → BMI  (ρ_1_) |  | BMI → UA  (ρ_2_) | P^a^ |  | RMR | CFI |
| Adult HTN group |  |  |  |  |  |  |  |
| NTN (n=390) | -0.016 |  | 0.089 | 0.143 |  | 0.04 | 0.86 |
| HTN (n=521) | 0.020 |  | 0.032 | 0.847 |  | 0.02 | 0.93 |
| P^b^ | 0.592 |  | 0.303 |  |  |  |  |
| Race |  |  |  |  |  |  |  |
| White (n=655) | -0.008 |  | 0.084* | 0.096 |  | 0.01 | 0.98 |
| Black (n=256) | 0.060 |  | 0.034 | 0.769 |  | 0.06 | 0.89 |
| P^b^ | 0.358 |  | 0.498 |  |  |  |  |
| Sex |  |  |  |  |  |  |  |
| Male (n=365) | 0.0002 |  | 0.064 | 0.390 |  | 0.03 | 0.91 |
| Female (n=546) | 0.021 |  | 0.069 | 0.428 |  | 0.02 | 0.92 |
| P^b^ | 0.759 |  | 0.941 |  |  |  |  |
| Follow-up years |  |  |  |  |  |  |  |
| 5~10 years (n=451) | 0.018 |  | 0.079 | 0.360 |  | 0.02 | 0.90 |
| 11~14 years (n=460) | -0.0005 |  | 0.047 | 0.472 |  | 0.01 | 0.95 |
| P^b^ | 0.781 |  | 0.629 |  |  |  |  |
| ρ =standardized regression coefficient; RMR=root mean square residual; CFI=comparative fit index; UA=uric acid; BMI=body mass index; NTN=normotension; HTN= hypertension (defined as SBP/DBP>=130/80 mmHg or taking medications)  a, p value for difference between ρ_1_ and ρ_2_  b, p value for difference between subgroups | | | | | | | |

| **Table S3.** Differences in effects of BMI and UA on SBP between race and sex groups | | | | |
| --- | --- | --- | --- | --- |
|  | Total  effect:  BMI→SBP  (c)^a^ | Indirect  effect 1:  BMI→UA  (β_1_) | Indirect  effect 2:  UA→SBP  (β_2_) | Direct  effect:  BMI→SBP  (c’)^b^ |
| Children |  |  |  |  |
| White (n=1838) | 0.376* | 0.387* | 0.074* | 0.347* |
| Black (n=1264) | 0.248* | 0.349* | 0.075* | 0.222* |
| P^d^ | 0.992 | 0.137 | 0.204 | 0.677 |
| Male (n=1601) | 0.307* | 0.377* | 0.055* | 0.286* |
| Female (n=1501) | 0.333* | 0.390* | 0.061* | 0.309* |
| P^d^ | 0.001 | <0.001 | 0.500 | 0.016 |
| Adults |  |  |  |  |
| White (n=2221) | 0.287* | 0.337* | 0.099* | 0.253* |
| Black (n=1181) | 0.208* | 0.302* | 0.120* | 0.172* |
| P^d^ | 0.569 | 0.204 | 0.262 | 0.464 |
| Male (n=1521) | 0.315* | 0.332* | 0.127* | 0.273* |
| Female (n=1881) | 0.220* | 0.407* | 0.080* | 0.188* |
| P^d^ | <0.001 | 0.675 | 0.166 | <0.001 |
| β, c and c’ are standardized regression coefficients.  BMI=body mass index; UA=uric acid; SBP=systolic blood pressure  a, BMI was not included in the models.  b, BMI was included in the models.  d, p value for difference between subgroups  *, P<0.05 for being different from 0 | | | | |


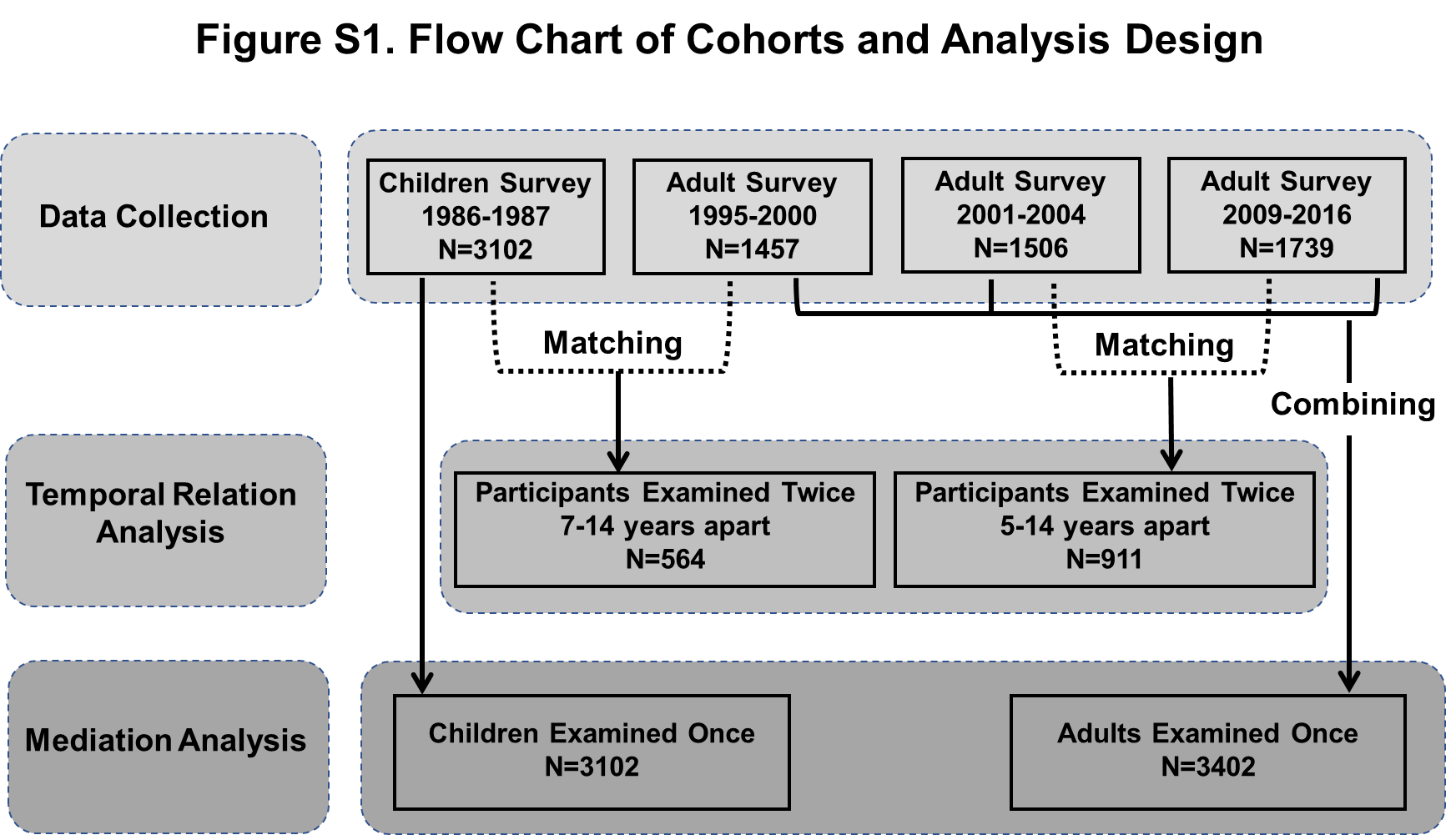


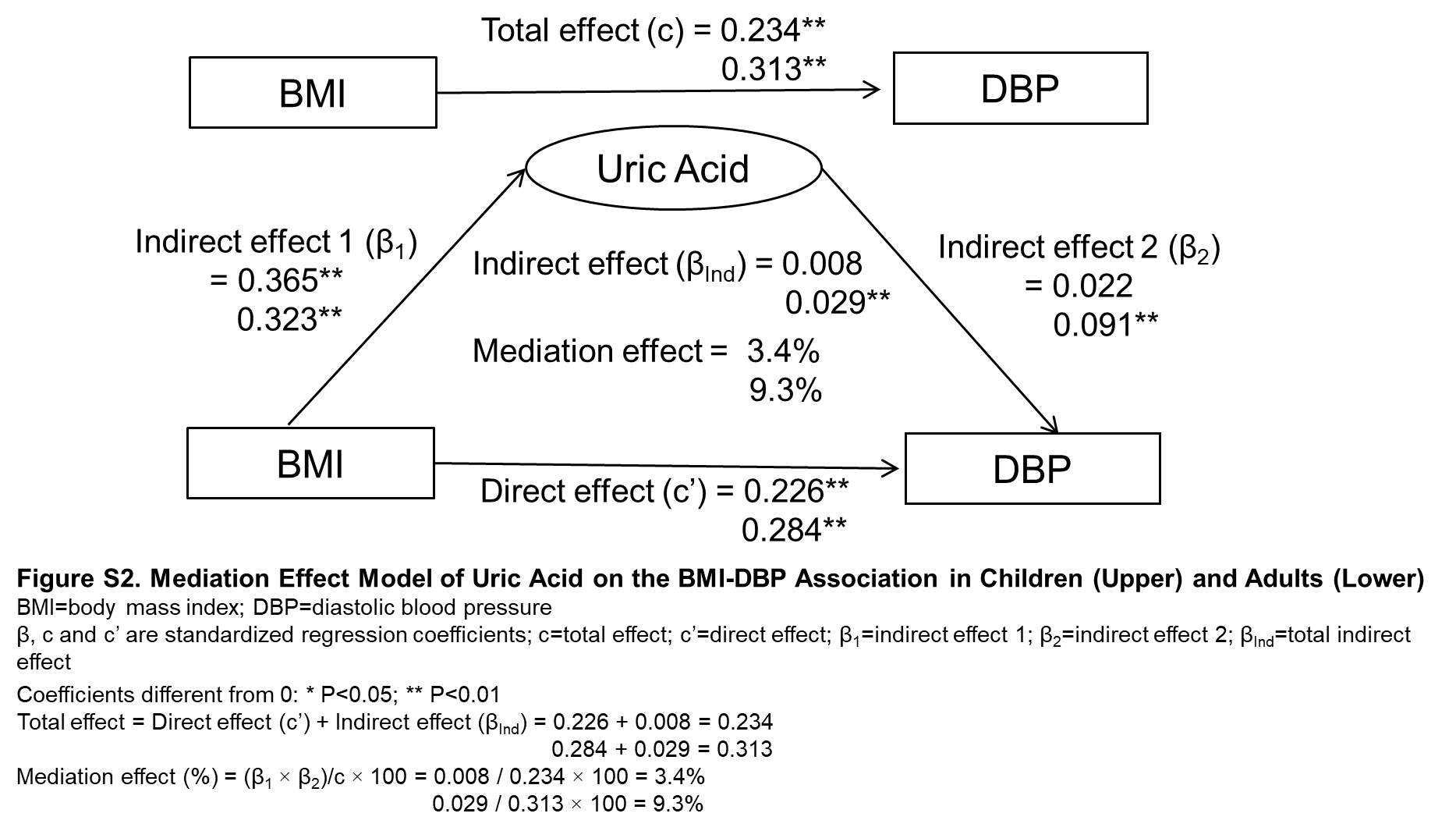


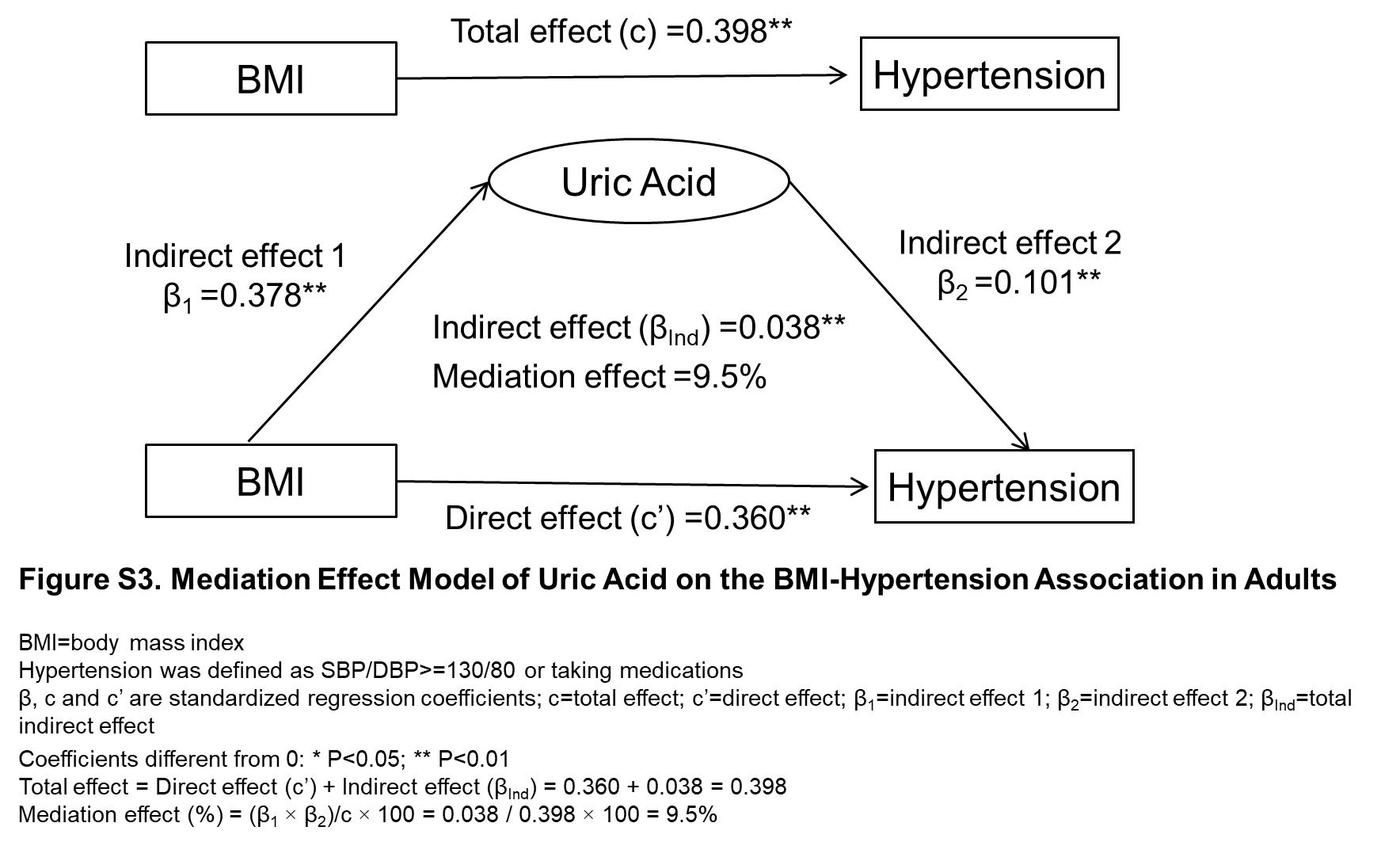

Supplement: Supplementary file 1 — Supplemental Materials [file 41366_2021_810_MOESM1_ESM.docx]
